# 166. Evaluation of Daptomycin Prescribing Practices Based on Microbiologic Susceptibility Determination of “Susceptible-Dose Dependent” (SDD)

**DOI:** 10.1093/ofid/ofab466.368

**Published:** 2021-12-04

**Authors:** Amy Rowley, Ashley H Marx, David J Weber, David J Weber

**Affiliations:** 1 University of North Carolina Eshelman School of Pharmacy, Raleigh, North Carolina; 2 University of North Carolina Medical Center, Durham, NC; 3 University of North Carolina, Chapel Hill, NC

## Abstract

**Background:**

Recent changes in CLSI microbiologic interpretations of daptomycin and enterococci include the “susceptible-dose dependent” (SDD) category. The effectiveness of SDD for directing clinicians to employ higher dosing of daptomycin is unknown. The study objective was to determine if implementation of SDD paired with a comment recommending higher doses of daptomycin (8-12mg/kg) and ID consultation in 2019 was associated with changes in rates of daptomycin use and prescribed doses for enterococcal bloodstream infections (BSI).

**Methods:**

Single-center, retrospective cohort study of adult inpatients with enterococcal BSI and daptomycin susceptibility results reported from Aug 2016-Jul 2020. Chart review was performed to collect demographics, source of infection, and clinical management strategy. Rate of daptomycin use for definitive therapy (antimicrobial on day 4 after final susceptibilities) and median prescribed dose were compared for BSI caused by S and SDD isolates. Annual (Aug 1-Jul 31) trends in infections and daptomycin use were tabulated.

**Results:**

189 blood cultures were reviewed, yielding 56 unique episodes of enterococcal BSI. Patients had a mean age of 59 years and majority had an immunocompromising disease or medication. Of the cases in which it was a clinically appropriate option, clinicians selected daptomycin for definitive treatment in 81% of S and 71% of SDD cases (p = 0.46, Chi-square). Median daptomycin dose prescribed was 10mg/kg for both interpretations; dose range was 6-12mg/kg for S and 9.5-12mg/kg for SDD isolates. No temporal trend in prescribed dose noted over the 4-year study period. Repeat blood cultures performed in 50/56 (89%). Within 90 days, rates of relapse were low but mortality was 26/56 (46%).

Table 1. Infection and Treatment Characteristics

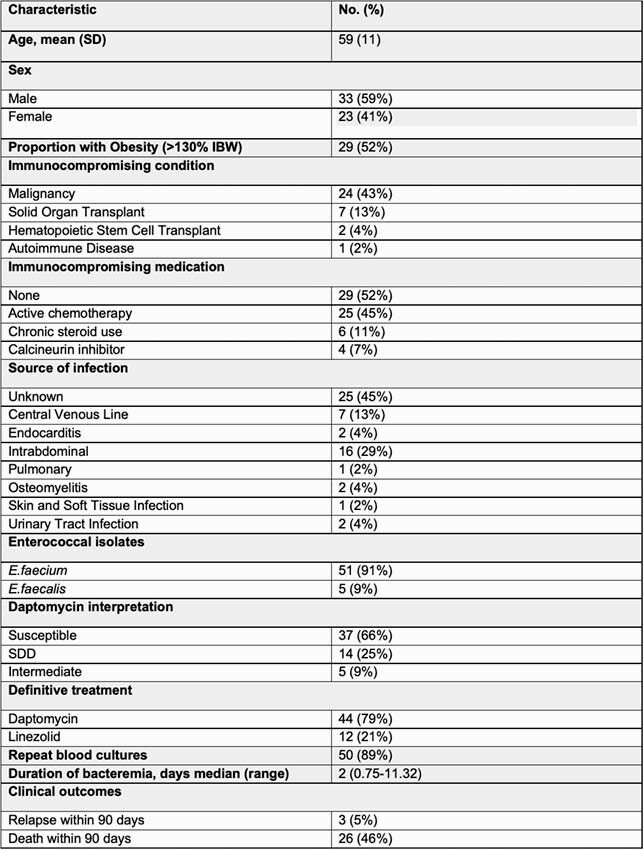

Chart 1. Frequency of prescribed daptomycin dose (mg/kg) for susceptible (A) and SDD (B) enterococci BSI isolates.

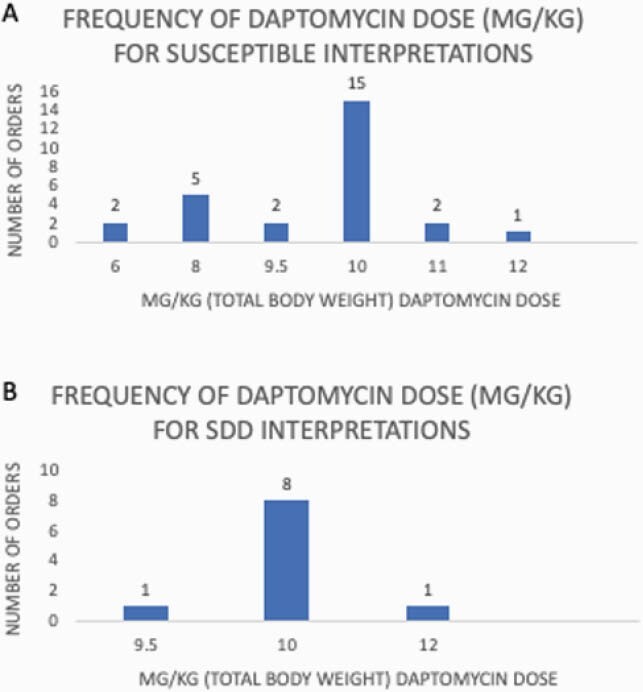

**Conclusion:**

No difference detected in rate of daptomycin use nor median prescribed dose based on microbiologic interpretation. While the majority of doses were adequate (10mg/kg) based on current guidance for enterococcal BSI, the use of a directive comment to guide dosing and ID consultation may have recused outliers. Additional data is needed to characterize the impact of specific microbiologic interpretations on clinician prescribing and determine the most effective messaging strategies.

**Disclosures:**

**David J. Weber, MD, MPH**, **PDI** (Consultant)

